# Catastrophic out-of-pocket payments for households of people with severe mental disorder: a comparative study in rural Ethiopia

**DOI:** 10.1186/s13033-019-0294-7

**Published:** 2019-06-01

**Authors:** Yohannes Hailemichael, Damen Hailemariam, Kebede Tirfessa, Sumaiyah Docrat, Atalay Alem, Girmay Medhin, Crick Lund, Dan Chisholm, Abebaw Fekadu, Charlotte Hanlon

**Affiliations:** 1 Department of Reproductive Health and Health Services Management, School of Public Health, College of Health Sciences, Addis Ababa University, Addis Ababa, Ethiopia; 2 Department of Psychiatry, School of Medicine, College of Health Sciences, Addis Ababa University, Addis Ababa, Ethiopia; 3 Centre for Global Mental Health, Health Service and Population Research Department, Institute of Psychiatry, Psychology and Neuroscience, King’s College, London, UK; 4 Alan J. Flisher Centre for Public Mental Health, Department of Psychiatry and Mental Health, University of Cape Town, Cape Town, South Africa; 5 Aklilu-Lemma Institute of Pathobiology, Addis Ababa University, Addis Ababa, Ethiopia; 6 Department of Mental Health and Substance Abuse, World Health Organization, Geneva, Switzerland; 7 Centre for Affective Disorders, Department of Psychological Medicine, Institute of Psychiatry, Psychology and Neuroscience, King’s College, London, UK

**Keywords:** Catastrophic health expenditure, Severe mental disorders, Low- and middle-income, Ethiopia, Universal health coverage

## Abstract

**Background:**

There are limited data on healthcare spending by households containing a person with severe mental disorder (SMD) in low- and middle-income countries (LMIC). This study aimed to estimate the incidence and intensity of catastrophic out-of-pocket (OOP) payments and coping strategies implemented by households with and without a person with SMD in a rural district of Ethiopia.

**Methods:**

A comparative cross-sectional community household survey was carried out from January to November 2015 as part of the Emerald programme (emerging mental health systems in low- and middle-income countries). A sample of 290 households including a person with SMD and 289 comparison households without a person with SMD participated in the study. An adapted and abbreviated version of the World Health Organization SAGE (Study on global Ageing and adult health) survey instrument was used. Households were considered to have incurred catastrophic health expenditure if their annual OOP health expenditures exceeded 40% of their annual non-food expenditure. Multiple logistic regression was used to explore factors associated with catastrophic expenditure and types of coping strategies employed.

**Results:**

The incidence of catastrophic OOP payments in the preceding 12 months was 32.2% for households of a person with SMD and 18.2% for comparison households (p = 0.006). In households containing a person with SMD, there was a significant increase in the odds of hardship financial coping strategies (p < 0.001): reducing medical visits, cutting down food consumption, and withdrawing children from school. Households of a person with SMD were also less satisfied with their financial status and perceived their household income to be insufficient to meet their livelihood needs (p < 0.001).

**Conclusions:**

Catastrophic OOP health expenditures in households of a person with SMD are high and associated with hardship financial coping strategies which may lead to poorer health outcomes, entrenchment of poverty and intergenerational disadvantage. Policy interventions aimed at financial risk pooling mechanisms are crucial to reduce the intensity and impact of OOP payments among vulnerable households living with SMD and support the goal of universal health coverage.

**Electronic supplementary material:**

The online version of this article (10.1186/s13033-019-0294-7) contains supplementary material, which is available to authorized users.

## Introduction

In a recent global monitoring report by the World Health Organization (WHO) and the World Bank, the incidence of catastrophic health expenditure was shown to have increased globally [1]. Indeed, each year, 100 million people face poverty and another 800 million people suffer due to catastrophic out-of-pocket (OOP) health expenditures [1]. Out-of-pocket payments make up a significant proportion of the total expenditure on mental health care in most low-income countries [2]. In 43% of countries in the African region, OOP spending is the main source of financing for mental health services [3].

In 2013/14, total health expenditure was approximately 4.7% of Ethiopian gross domestic product (GDP), with per capita health expenditure amounting to US$28.65 [4]. Household out-of-pocket expenditure represents the largest financing source for health care, accounting for about 33% of total health expenditure [4]. Although a fee waiver program was introduced as a measure to protect the poor from the negative effects of out-of-pocket payments in Ethiopia, the coverage remains low in many regions [5]. Hence, chronic illnesses with ongoing need for care, as is seen with severe mental disorders (SMD) such as schizophrenia and bipolar disorder, exert a heavy economic burden on vulnerable households in accessing mental healthcare [6]. Evidence indicates that the extent of out-of-pocket payments for mental health problems varies from country to country and according to the methodology used [7–9]. In an Indian study, the percentage of women with severe depression who had experienced catastrophic expenditure was 14.6%, compared to 4.9% for the non-cases [7]. Similarly, in a study conducted in Bangladesh, 15.1% of households with a person with mental illness encountered increased household expenditure that resulted in distress financing [8].

Out-of-pocket health care spending imposes a substantial financial burden on households in many countries and leads the household to sacrifice consumption of other items, to incur debts, or to sell productive resources [10, 11]. Furthermore, health care expenditures have been reported as one of the major pathways into poverty and an important reason why many households remain poor [12–15].

Although studies have reported the impact of out-of-pocket expenditure on households; the investigation of household costs of disease is incomplete if coping strategies employed by households are ignored [16, 17]. Evidence suggests that in most African countries, the health financing system is too weak to protect households from health shocks (illness). Hence, using coping strategies to finance health care is common [18]. These strategies include informal transfers (in cash and in-kind) from relatives and friends [19], selling assets [20] or obtaining loans [14, 21], withdrawing children from school [20, 22] and taking on extra work [23]. Evidence from middle-income Asian countries, including China [24], Vietnam [25, 26], and Thailand [27], indicates that illness and associated medical expenditures have a significant negative impact on household basic consumption. In the Ethiopian context, Asfaw and Braun [28] and Dercon et al. [29] show that when the head of the household is the person who is unhealthy, non-medical consumption declines sharply, with reductions ranging from 15 to 35%.

The study reported in this paper is part of the cross-country Emerald programme (emerging mental health systems in low- and middle-income countries) which sought to provide rigorous, population-based evidence about the adequacy and fairness of mental healthcare financing [30].

In this study, we investigated health expenditure and financial coping strategies in relation to SMD in rural Ethiopia. We hypothesized that the incidence and intensity of catastrophic health expenditure and associated coping strategies would be significantly higher in households including a person with SMD compared to households with no affected family member. We also explored the factors associated with catastrophic health expenditure and coping strategies.

## Methods

### Study design, setting, and participants

A comparative cross-sectional household survey was carried out in Sodo district, in southern Ethiopia. Administratively, Sodo is structured into 58 sub-districts (*kebeles*) with a total population of 161,952, of which 90% resides in rural areas [31]. At the time of this study, primary health care was provided in eight health centers and in 58 health posts. There are also a number of private clinics and drug vendors throughout the district [32]. Sodo district was the site for a model of primary care-based mental healthcare, through the PRogramme for Improving Mental health carE (PRIME) [33]. The Emerald programme was linked to PRIME, with the overall aim of investigating the health system interventions required to support integrated primary mental health care. The current study was conducted as part of Emerald at the baseline of PRIME, prior to implementation of mental healthcare. At that time, there was no mental health service available in the district. Psychiatric nurse-led out-patient care was available 30–50 km away and the closest in-patient mental health care was available in the capital city, Addis Ababa (100 km away). However, the SAGE questionnaire also included OOP costs for non-specialist health care, conceptualized broadly to also include care from religious and traditional healers.

Community-based identification of people with possible SMD (n = 467) was conducted by community-based health extension workers and community leaders who had received half a day of training on common presentations of SMD for the setting [33]. This approach was used previously in the nearest district and found to be effective [34]. Referred cases were screened by a primary care worker based at the local health center. The primary care workers (nurses, health officers and midwives) had been trained using the World Health Organization mental health Gap Action Programme (mhGAP) [35]. The primary care workers followed the mhGAP intervention guide to make diagnoses of psychosis or bipolar disorder (n = 294). A confirmatory clinical diagnosis of SMD (any psychotic disorder or bipolar disorder) (n = 300) was then made by psychiatric nurses trained to administer the operational criteria for research (OPCRIT) semi-structured clinical interview [36].

For this sample, the comparison group (n = 289) of households of persons without SMD who fulfilled the matching criteria was randomly selected from a sampling frame based on a household census of the district which was conducted by PRIME [37]. Households of a person with SMD were matched to comparison households without a person with SMD on the basis of age of the household head, gender, *gott* (lowest residential administrative area) and household size. The eligibility criteria were: age 18 years and above, planned to reside in the area for the next 12 months (to participate in the longitudinal study) and able to comprehend the interview. Recruitment took place from January to November 2015.

### Data collection, definitions, and measurements

Outcome data were collected using an adapted and abbreviated version of the World Health Organization SAGE (Study on global Ageing and adult health) survey instrument [38]. The instrument was used to collect information on: (i) household characteristics (sex, age, schooling, marital status, residence); (ii) housing characteristics (type, building materials, water, sanitation and ownership); (iii) household consumption expenditures (collected item by item); and (v) household and family support networks and transfers, including financial/non-financial help from family and friends. Different reference periods were used for each type of expenditure: 7 day, 30 day and 12 month recall periods prior to the date of interview. The household questionnaire was administered to the household head by trained and experienced data collectors. In the absence of the household head the most knowledgeable member of the household about the household consumption was interviewed.

Out-of-pocket payment for health care includes all categories of expenditures for consultation, investigations, medication and other procedures or interventions, for all types of healthcare, including biomedical, traditional and religious healing with a 1 month recall period, and the costs of infrequent health care (i.e. hospital admissions) over a 12 month recall period. Household health expenditures were converted and summed to obtain the annual cost and then expressed as a percentage of annual household total consumption expenditure and non-food consumption expenditure at various threshold levels.

The primary outcome (incidence of catastrophic out-of-pocket payment for health care) was measured as the percentage of households incurring health payments in excess of a specified threshold in 1 year [39]. The intensity of catastrophic costs measures the extent to which households exceeded the chosen threshold [40]. Overshoot, which equals the difference between the estimated out-of-pocket payment as a share of total consumption expenditure or capacity to pay and the threshold among all individuals; and the mean positive overshoot, which indicates the overshoot among the subgroup of those who exceeded the threshold [40]. However, the threshold levels considered for calculation of catastrophic payments may differ according to country context [11, 41]. Therefore, a sensitivity analysis was performed using various thresholds (i.e. 5%, 10%, 15%, 20%, 25% and 40%) of household total consumption expenditure and household capacity to pay. In the literature the threshold of what constitutes catastrophic expenditure is debatable. The most widely accepted method in the literature and recommended by the World Health Organization and Xu et al. [11] for measuring the incidence of catastrophic payments is capacity to pay [42]. Capacity to pay is defined as payment on non-discretionary or non-subsistence spending (roughly, non-food expenditure). We estimate health expenditure to be catastrophic when it exceeds ≥ 40% of non-food expenditure.

The choice of the threshold level is based on the idea that households will be left with a certain balance of their pre-expenditure income or capacity to pay that would allow them to spend on other needs in the household [40, 43].

With regard to coping strategies (the secondary outcome), the survey included questions on the strategies that the household had employed in response to financial distress over the previous year. Such strategies included the following: cost minimization strategies (i.e. withdrawing children from school, reducing food consumption, reducing frequency of medical visits); and cost management strategies (i.e. use of savings, finding extra work, taking out loans from a bank or money lender, drawing up accounts at shops, and asking friends and relatives for help) [10].

Functional disability in the previous month for the index patient and for the interviewed household member in the comparison group was assessed using the 36-item World Health Organization Disability Assessment Schedule-II (WHODAS-II), which has been previously validated in a rural Ethiopian setting [44].

### Statistical analysis

The data were double-entered using Epidata 3.1 [45] and cleaned and analyzed using STATA version 13.1 [46]. The two-sample t test, the non-parametric Mann–Whitney test and Chi-square test-for-trend were used to compare means, medians and proportions across households with a person with SMD and households without the disorder. Chi-square test was used to examine bivariate association of mental health status with catastrophic payment at the 5%, 10%, 15%, 20%, 25% and 40% thresholds. The crude and adjusted association of different factors with catastrophic health payment based on capacity to pay (≥ 40% of household non-food consumption) were estimated with odds ratios, 95% confidence intervals and p-values, using logistic regression. Households that incurred health payments in the previous year and risk factors were included in the regression model.

Logistic regression models were fitted to examine the independent effect of having a person with SMD in the household on catastrophic expenditure and to explore the factors associated with a greater likelihood of implementing particular coping strategies. The model was run separately for each coping strategy using the same set of independent variables. Odds ratios and 95% confidence intervals were used as the measure of the effect size.

## Results

A flow diagram of participant recruitment and inclusion in the study is presented in Additional file 1: Figure S1.

### Household socio-demographic and economic characteristics

The study enrolled 290 households including persons with SMD, with a total of 1513 household members, and 289 households of a person without a person with SMD, with a total of 1522 household members. As shown in Table 1, the mean age of the household head was nearly 50 years in both groups. The mean number of adults per household was higher in households of a person with SMD than in comparison households (3.2 vs. 2.9; p = 0.004). A higher proportion of head of households of a person with SMD were never married (p = 0.007). The proportion of households covered by health insurance was lower for households having a member with SMD compared to households without a person with SMD (1.0% vs. 2.7%; p = 0.262). The mean number of children younger than 15 years for households of a person with SMD was considerably lower than for comparison households (p = 0.004). Households of a person with SMD reported higher levels of disability (p < 0.001) and lower median (inter-quartile range; IQR) expenditure on annual food consumption (Birr 6087.8; IQR 3785.5, 9533.3), than households without a person with SMD (Birr 6240.0; IQR 4356.7, 10,111.1).

**Table 1:** Background characteristics of households

Household characteristics	Households of person with severe mental disorder (SMD) (n = 290)	Comparison households without person with SMD (n = 289)	p-value
Socio-demographic and economic			
Household members (N)	1513	1522	
Household size, mean (SD)	5.2 (2.2)	5.3 (2.1)	0.508
Household composition, mean (SD)^a^	2.8 (0.9)	2.7 (0.8)	0.915
Mean number of adults in household	3.2 (1.3)	2.9 (1.1)	0.004
Children younger than 15 years, mean (SD)	1.9 (1.7)	2.3 (1.6)	0.004
Residence, n (%)			
Urban	55 (18.9)	53 (18.3)	0.847
Rural	235 (81.0)	236 (81.7)	
Gender, n (%)			
Male	210 (72.7)	223 (78.0)	0.140
Female	79 (27.3)	63 (22.0)	
Head age (years), mean (SD)	49.5 (14.2)	49.9 (14.0)	0.867
Head marital status, n (%)			
Never married	20 (6.9)	5 (1.7)	0.007
Married	205 (70.9)	223 (77.7)	
Separated/divorced/widowed	64 (22.1)	59 (20.5)	
Head education, n (%)			
No formal education	185 (63.7)	179 (61.9)	0.788
Primary education	76 (26.2)	83 (28.7)	
More than primary	29 (10.0)	27 (9.3)	
Head with health insurance, n (%)	3 (1.0)	8 (2.7)	0.262
Annual median (IQR) food consumption^b^	6087.8 (3785.5, 9533.3)	6240.0 (4356.7, 10,111.1)	0.868
Annual median (IQR) non-food consumption^b^	1570.0 (744.2, 3179.1)	1627.5 (680.0, 3235.5)	0.803
Annual median (IQR) health consumption^b^	290.9 (166.6, 678.2)	355.5 (195.2, 733.3)	0.308
Consumption quintile			
Lowest	62 (21.4)	52 (17.9)	0.613
Low	64 (22.1)	57 (19.7)	
Middle	54 (18.6)	63 (21.8)	
High	54 (18.6)	63 (21.8)	
Highest	55 (19.0)	54 (18.6)	
Clinical characteristics			
WHODAS, median (IQR)	52.7 (31.9, 69.4)	5.5 (0.0, 19.4)	0.000

WHODAS score for the index patient and for the interviewed household member in the comparison group

WHODAS: World Health Organization Disability Assessment Schedule; IQR: inter-quartile range; SD: standard deviation

^a^Adult equivalent

^b^Birr; US$1 = Birr 20.69 (2015)

### Out-of-pocket (OOP) health expenditure

Of the 290 households with SMD 83.1% (n = 241) and out of 289 households without SMD, 39.7% (n = 115) had accessed and received services in terms of consultations, medications, investigations, traditional healing, hospitalization and other services in the previous 1 month and 12 months preceding the day of the data collection.

OOP payments as a share of total consumption were not significantly higher for households of a person with SMD than in comparison households (6.9% vs. 5.7%). The percentages of health payments spent on medication (74.1% vs. 71.1%), investigations (29.6% vs. 26.4%), and consultations (16.7% vs. 16.3%) were non-significantly higher for households of a person affected by SMD compared to households without a person with SMD (Table 2).

**Table 2:** Household expenditure categories

Consumption category	Households of person with SMD (n = 290)		Comparison households without person with SMD (n = 289)		p-value
	%	95% CI	%	95% CI	
% of total consumption^a^					
All food items	75.4	(73.5, 77.4)	77.0	(75.1, 79.0)	0.202
Housing and livelihood regular expenses	15.2	(13.5, 16.9)	15.7	(13.9, 17.4)	0.243
Big household expenditures	3.1	(1.7, 4.6)	3.4	(1.6, 6.1)	0.351
Health expenditures	6.9	(5.8, 8.0)	5.7	(4.5, 6.9)	0.151
% of health payments					
Consultation	16.7	(13.9, 19.4)	16.3	(11.3, 21.3)	0.475
Medication	74.1	(70.7, 77.4)	71.1	(65.8, 76.5)	0.427
Investigations	29.6	(23.2, 36.0)	26.4	(20.5, 32.4)	0.846

Food items (staple foods, vegetable, fruit, spices etc.), regular household expenses (electricity, water, cooking, renting, clothing, transport, etc.), Big expenditure include (education, durable goods, cultural ceremonies, entertainment, tax etc.); health expenditure include expense on (outpatient consultation, medication, investigations, hospitalization, medical appliances, ambulance, etc.)

^a^Adult equivalent

### Incidence and intensity of catastrophic payments

With the threshold set at 10% of total consumption expenditure, 20.3% (95% CI 15.6, 25.9) of households of a person with SMD and 15.6% (95% CI 10.0, 23.5) of comparison households experienced catastrophic payments (Table 3). With the threshold set at 25% of household total consumption, the same trend emerged, whereby 4.6% among households of a persons with SMD compared to 2.7% among comparison households incurred catastrophic health expenditure.

**Table 3:** Incidence and intensity of catastrophic expenditure for different thresholds

Sample	Threshold						Overshoot at 10%	Mean positive overshoot at 10%
	5%	10%	15%	20%	25%	40%		
As a proportion of total consumption								
HH of a person with SMD	42.7	20.3	16.5	12.1	4.5	1.2	1.9	9.7
HH without a person with SMD	41.7	15.6	15.4	12.0	2.6	0.0	1.2	7.6

Sample	Threshold						Overshoot at 40%+	Mean positive overshoot at 40%+
	5%	10%	15%	20%	25%	40%		
As a proportion of capacity to pay								
HH of a person with SMD	89.6*	74.3	65.7	62.2	48.3	32.2**	6.7*	21.2
HH without a person with SMD	80.8	65.2	57.5	52.3	38.2	18.2	2.9	15.9

HH: household

* p < 0.05; ** p < 0.01

At a threshold level of 40%+ of non-food expenditure (capacity to pay), the level of catastrophic payments was 32.2% (95% CI 26.6, 38.4) for households of a person with SMD and 18.2% (95% CI 12.1, 26.4) for comparison households; Pearson Chi^2^ (1) 7.592, p = 0.006.

The proportion of mean positive overshoot (MPO) at the 40% threshold level was as high as 21.2% for households of a person with SMD. In comparison households, the proportion was 15.9%; p = 0.230 (Table 3).

### Determinants of catastrophic expenditure and coping strategies

In the multivariable model, the odds of experiencing catastrophic OOP health payments were significantly higher for households of a person with SMD (adjusted odds ratio (aOR 1.5; 95% CI 1.0, 2.7; p = 0.041), households in the lowest consumption quintile (aOR 10.8; 95% CI 3.8, 30.5; p < 0.001) compared to those in the highest quintile, and in households with a member aged 60 years and above (aOR 1.2; 95% CI 1.1, 2.2; p = 0.047). The odds of incurring catastrophic OOP health payments was lower for households with more members (aOR 0.6; 95% CI 0.4, 0.9; p = 0.021). There was no significant difference in the odds of incurring catastrophic health payments with respect to area of residence, gender and educational status of the head of the household (Table 4).

**Table 4:** Factors associated with catastrophic health payments at the 40%+ threshold capacity to pay among households with a person with SMD and comparison households

Factors	Monthly OOP payment in Birr, mean (SD)	Share of health payment as % of non-food consumption	OOPCHE (40%+)	Unadjusted model	Adjusted model
				Odds ratio (95% CI)	Odds ratio (95% CI)
Households of person with SMD	50.6 (82.1)	30.6	32.2	2.1 (1.2, 3.6)**	1.5 (1.0, 2.7)*
Households without person with SMD	51.7 (61.0)	22.7	18.2	1.00^a^	1.00^a^
Household size				0.5 (0.4, 0.7)***	0.6 (0.4, 0.9)**
Age ≥ 60 years in HH					
Yes	50.0 (78.8)	29.1	33.0	1.4 (0.9, 2.3)	1.2 (1.1, 2.2)*
No	50.5 (64.3)	27.2	25.1	1.00^a^	1.00^a^
Area of residence					
Urban	58.1 (74.2)	26.6	27.1	0.9 (0.5, 1.7)	1.3 (0.6, 2.5)
Rural	49.2 (76.3)	28.4	27.8	1.00^a^	1.00^a^
Gender of the head of the HH					
Male	49.9 (75.5)	26.7	25.4	0.6 (0.3, 1.1)	0.9 (0.5, 1.7)
Female	50.9 (71.3)	31.0	34.0	1.00^a^	1.00^a^
Household head education					
No formal education	44.2 (54.3)	27.1	24.3	0.8 (0.3, 1.8)	0.7 (0.3, 1.9)
Primary education	63.0 (106.0)	29.8	35.3	1.4 (0.6, 3.2)	1.5 (0.6, 3.9)
More than primary education	59.8 (88.4)	28.6	27.7	1.00^a^	1.00^a^
Children in the household					
0	54.8 (72.0)	36.0	40.5	2.6 (1.4, 4.7)**	1.2 (0.5, 3.0)
1–2	56.9 (73.3)	29.0	27.8	1.4 (0.8, 2.5)	0.8 (0.4, 1.7)
3 and more	43.5 (80.0)	22.8	20.6	1.00^a^	1.00^a^
Consumption quintile					
Quintile 1 (lowest)	21.2 (14.3)	38.0	45.7	9.5 (3.7, 24.0)***	10.8 (3.8, 30.5)***
Quintile 2	34.5 (32.4)	34.8	38.0	6.9 (2.7, 17.5)***	8.2 (2.9, 22.9)***
Quintile 3	59.7 (79.4)	29.6	29.3	4.6 (1.8, 11.7)**	5.7 (2.0, 15.8)**
Quintile 4	60.1 (70.0)	26.7	25.6	3.8 (1.5, 9.9)**	4.9 (1.7, 13.5)**
Quintile 5 (highest)	67.7 (110.5)	16.0	8.1	1.00^a^	1.00^a^

OOPCHE: out-of-pocket catastrophic health expenditure; HH: household

* p < 0.05; ** p < 0.01; *** p < 0.001; US$1 = Birr 20.69 (2015)

^a^Reference group

### Household satisfaction in livelihood, financial difficulties and coping

As shown in Table 5, only 8.7% (95% CI 5.1, 12.3) of households of a person with SMD were satisfied with their financial status compared with 19.1% (95% CI 11.8, 26.4; p = 0.005) among households with no person with SMD. In households of a person with SMD, just 10.3% (95% CI 6.4, 14.2) perceived their household income to be enough to meet their livelihood needs, compared to 24.3% (95% CI 16.3, 32.3; p = 0.001) in households without a person with SMD. Households of a person with SMD were also less likely to be satisfied with their livelihoods (p < 0.001).

**Table 5:** Household satisfaction in livelihood, financial difficulties and coping

Financial difficulties and coping	Households with out-of-pocket health payments				
	Households of person with SMD (n = 241)		Households without person with SMD (n = 115)		p-value
	No	% (95% CI)	No	% (95% CI)	
Household financial situation and livelihood					
Perceive income is enough	25	10.3 (6.4, 14.2)	28	24.3 (16.3, 32.3)	0.001
Satisfied with financial situation	21	8.7 (5.1, 12.3)	22	19.1 (11.8, 26.4)	0.005
Satisfied with livelihood	89	36.9 (30.7, 40.0)	71	61.7 (52.7, 70.7)	0.000
Coping strategies implemented					
Drew up accounts at shops	71	29.4 (23.6, 35.2)	26	22.6 (14.8, 30.3)	0.175
Loan from bank/financial institution	57	23.6 (18.2, 29.0)	25	21.7 (14.0, 29.3)	0.689
Cut down food consumption	105	43.5 (37.2, 49.8)	20	17.3 (10.3, 24.4)	0.000
Reduced medical visits	68	28.2 (22.4, 33.9)	7	6.0 (1.6, 10.5)	0.000
Asked help from community	25	10.3 (6.4, 14.2)	6	5.2 (1.0, 9.3)	0.107
Asked help from family	93	38.5 (32.3, 44.7)	25	21.7 (14.0, 29.3)	0.002
Withdrew children from school	56	23.2 (17.8, 28.6)	9	7.8 (2.8, 12.8)	0.000
Took on extra work	92	38.1 (31.9, 44.3)	26	22.6 (14.8, 30.3)	0.004
Used savings	23	9.5 (5.8, 13.2)	12	10.4 (4.7, 16.1)	0.792
Sold assets	152	66.3 (60.2, 72.5)	86	80.3 (72.7, 88.0)	0.009
More than one strategy	159	65.9 (59.9–71.9)	45	39.5 (30.0–48.1)	0.000

See Additional file 2: Table S1 for unadjusted and Table 6 for adjusted multivariable logistic regression results on coping strategies that household employed to respond to financial stress. The sale of assets was a common strategy in the study population. However, households with a person with SMD were less likely to sell assets (aOR 0.4; 95% CI 0.2, 0.7; p = 0.009) compared to the households without a person with SMD.

**Table 6:** Adjusted odds of coping strategies for financial difficulties by mental health disorder and covariates

Characteristics	Total by subgroup	Coping strategies implemented for financial constraint							
		Sold assets (n = 238)	Drew up accounts at shops (n = 97)	Cut down food consumption (n = 125)	Withdrew children from school (n = 65)	Relatives/family assistance (n = 118)	Reduce medical visits (n = 75)	Used savings (n = 35)	Took on extra work (n = 118)
		AOR (95% CI)	AOR (95% CI)	AOR (95% CI)	AOR (95% CI)	AOR (95% CI)	AOR (95% CI)	AOR (95% CI)	AOR (95% CI)
HH of person with SMD	241	0.4 (0.2, 0.9)*	1.5 (0.8, 2.7)	3.3 (1.8, 6.0)***	3.1 (1.4, 6.7)**	2.4 (1.4, 4.1)**	5.6 (2.4, 13.0)***	0.8 (0.3, 1.8)	1.8 (1.1, 3.2)*
HH without person with SMD	115	1.00^a^	1.00^a^	1.00^a^	1.00^a^	1.00^a^	1.00^a^	1.00^a^	1.00^a^
Residence									
Urban	70	0.08 (0.04, 0.17)***	3.0 (1.6, 5.4)***	1.1 (0.6, 2.1)	0.9 (0.4, 1.9)	0.9 (0.5, 1.7)	1.0 (0.4, 2.1)	0.7 (0.2, 2.2)	2.4 (1.3, 4.4)**
Rural	286	1.00^a^	1.00^a^	1.00^a^	1.00^a^	1.00^a^	1.00^a^	1.00^a^	1.00^a^
Gender									
Male	262	1.4 (0.8, 2,7)	0.6 (0.3, 1.1)	0.4 (0.2, 0.8)**	1.1 (0.5, 2.1)	1.1 (0.6, 1.9)	0.4 (0.1, 0.7)**	1.3 (0.5, 3.4)	1.3 (0.8, 2.3)
Female	91	1.00^a^	1.00^a^	1.00^a^	1.00^a^	1.00^a^	1.00^a^	1.00^a^	1.00^a^
Household consumption									
Quintile 1 (lowest)	58	0.6 (0.2, 1.5)	1.7 (0.7, 4.0)	4.1 (1.8, 9.2)**	1.9 (0.7,5.0)	1.0 (0.4, 2.2)	10.2 (3.7, 27.9)***	1.8 (0.5, 5.6)	2.6 (1.2, 5.8)*
Quintile 2	63	0.7 (0.2, 1.6)	1.1 (0.5, 2.5)	2.6 (1.1, 5.7)*	1.8 (0.7, 4.6)	0.9 (0.4, 1.9)	4.9 (1.7, 13.6)**	1.0 (0.3, 3.5)	2.9 (1.3, 6.2)**
Quintile 3	75	0.5 (0.2, 1.2)	1.4 (0.6, 3.0)	3.2 (1.5, 7.0)**	1.6 (0.6, 4.0)	1.4 (0.7, 2.8)	6.9 (2.4, 19.2)***	1.3 (0.4, 3.8)	2.3 (1.1, 4.8)*
Quintile 4	74	0.9 (0.4, 2.2)	1.6 (0.7, 3.3)	1.5 (0.6, 3.3)	1.2 (0.4, 3.1)	1.2 (0.6, 2.4)	5.0 (1.7, 14.1)**	1.1 (0.3, 3.6)	1.8 (0.8, 3.7)
Quintile 5 (highest)	86	1.00^a^	1.00^a^	1.00^a^	1.00^a^	1.00^a^	1.00^a^	1.00^a^	1.00^a^

The model was run separately for each coping strategy using the same set of independent variables

CI: 95% confidence interval; AOR: Adjusted odds ratio; HH: household

* p < 0.05, ** p < 0.01, *** p < 0.001

^a^Reference group

The odds of coping by reducing food consumption (aOR 3.3; 95% CI 1.8, 6.0; p < 0.001), withdrawing children from school (aOR 3.1; 95% CI 1.4, 6.7; p = 0.003), obtaining assistance from relatives (aOR 2.4 95% CI 1.4, 4.1; p = 0.001), reducing the frequency of medical visits (aOR 5.6; 95% CI 2.4, 13.0; p < 0.001) and taking on extra work (aOR 1.8; 95% CI 1.1, 3.2; p = 0.019) were higher for households of a person with SMD than comparison households (Table 6).

Households in the lowest quintile for expenditure had the highest odds of coping through reduction of food consumption (aOR 4.1; 95% CI 1.8, 9.2; p = 0.001) and medical visits (aOR 10.2; 95% CI 3.7, 27.9; p = 0.001) compared to the wealthiest group (Table 6).

Urban households were more likely to draw up accounts at shops (aOR 3.0; 95% CI 1.6, 5.4; p < 0.001), households in the lowest wealth quintile had greater odds of taking on extra work (aOR 2.4; 95% CI 1.3, 4.4; p = 0.014) and male household heads were significantly less likely to experience consumption reduction (aOR 0.4; 95% CI 0.2, 0.8; p = 0.009) and interrupt medical visits (aOR 0.4; 95% CI 0.1, 0.7; p = 0.008), respectively (Table 6).

## Discussion

In our study, the incidence rate of catastrophic health expenditure at various thresholds of household total expenditure and capacity to pay was consistently higher for households of a person with SMD compared to households without a person with SMD. In both households with and without SMD, the level of catastrophic health spending was high compared to the average for the African region of 11.4% [47]. Furthermore, at a 25%+ threshold of total expenditure, the proportion of households experiencing catastrophic expenditure was almost two times higher for households with SMD compared with households without an affected person. Based on the threshold of 40%+ of consumption-based capacity to pay, our result remains significantly higher for households of a person with SMD.

Similarly, Zuvekas and Selden [48] in USA, Patel et al. [7] in India and Zergaw et al. [49] in Ethiopia reported that families with one or more member experiencing mental health problems were more likely to have high out-of-pocket spending compared with the control groups. Moreover, this study is consistent with findings from an earlier study from the USA that reported people with bipolar disorder incurred over four times greater costs compared with the non-bipolar group [50]. Likewise, in São Paulo [51] and in the USA [52], having a mental disorder was strongly correlated with higher health care payments. The higher burden of catastrophic health payments in households of a person with SMD can be explained by the financing structure of the available health system, the scarcity of locally available treatment services, and the marked disabling nature of the disorder that may increase health service use which in turn leads to high OOP expenditure.

We found evidence of regressive health spending, whereby households in the lowest quintile of socio-economic status paid 38.0% of their capacity to pay compared to only 16.0% for the highest quintile. The differences found for catastrophic health spending based on capacity to pay in relation to mental health status were significant and also followed a pattern linked to socio-economic status of the household. These results indicate that catastrophic health spending affects more households of a person with SMD and the poorest households. In the absence of financial protection (fewer than 3% of households in our study were enrolled in a social protection scheme), catastrophic out-of-pocket payments will further increase the number of households of a person with SMD falling into poverty, as well as potentially act as a deterrent from using services. Having an older household member was also associated with catastrophic health expenditure. In other studies, the presence of elderly members in the household was also found to lead to catastrophic health expenditure [53–55]. This may be because older adult members require more health care that may result in higher health expenditure. Nonetheless, there was no statistically significant difference between gender, educational level of the head of the household, residential location, children in the household and incurring catastrophic health expenditure. Previous studies on non-mental health problems reported mixed findings [53, 56–59].

This study has shown that mental health status, demographic and economic factors play an important role in the coping strategy adopted by households in Sodo. Our study indicated that the probability of withdrawing children from school was significantly higher for households of a person with SMD. A number of studies have found that children are taken out of school during times of illness in order to take up the slack in the workload in their families [12, 20, 22, 60]. A recent population-based cohort study in Ethiopia demonstrated that maternal common mental disorder was associated with child school dropout and absenteeism [61]. For people with SMD, the impact on child education may be more enduring due to the tendency for the illness to persist or recur. From an economic point of view, such action in response to healthcare costs will have a long-term impact on investment in human capital which may have serious implications for the education of future generations.

We had shown previously in the same sample that households of people with SMD are more likely to be severely food insecure than comparison households [62]. Our finding that households of a person with SMD were more likely to reduce their daily food consumption to cope with healthcare costs indicates that catastrophic health costs are one pathway towards food insecurity. When OOP payments comprise a large share of household budgets, households are at risk of sacrificing current consumption and experience food insecurity to pay for medical costs [60, 63–65]. Previous study on coping strategies for cost of illness in Ethiopia found that roughly one-third of households had to sacrifice other essential spending to pay for medical treatment [23]. Therefore, programmes that reduce out-of-pocket payments may indirectly improve access to food and nutrition.

Severe mental disorders are often chronic or recurrent, with a need for ongoing medical care for the best outcome. However, the results indicate that households of a person with SMD were more likely to adopt cost prevention strategies, like reducing medical visits, compared to comparison households, at the time when they most needed to access services. This strategy may threaten the person’s recovery from illness. Studies on non-communicable diseases reported similar findings [60, 66, 67].

Selling of assets in households of a person with SMD tended to be significantly lower than comparison households. This is explained by the possession of fewer assets that could be sold by these households [68]. Likewise, despite greater need, borrowing or taking a loan was not a common strategy adopted by households of a person with SMD. This might be because households with a person with SMD have less capacity to get loans because they cannot guarantee repayments. Nonetheless, previous studies on coping strategies for cost of illness in Ethiopia have found that roughly one-third had to adopt a cost management strategy, such as borrowing, selling assets or mortgaging a crop [23, 69]. Empirical evidence shows that borrowing and selling assets to cope with medical costs ranged from 23% of households in Zambia to 68% in Burkina Faso [18]. In India, half of the out-of-pocket expenditure made by households for psychiatric disorders came from loans [70]. This is not in line with our findings. Studies in the literature reported that coping strategies that can be adopted for financial stress are likely to be context and country specific [71, 72].

A transfer of either goods/money from friends and relatives was reported to be a common strategy in households of people with SMD in our study. Empirical evidence from low-and middle-income countries indicates that assistance from relatives and friends is very common when household members face illness, and that this acts as an insurance mechanism rather than as credit [73].

The least common coping response by households of a person with SMD to the financial stress in our data was the use of savings. This may reflect the fact that households of a person with SMD rarely have savings. Gresenz and Sturm [74] found that, compared to mentally healthy individuals, mentally ill individuals are 75% less likely to have any savings.

## Strengths and limitations

Our study is a population-based study of people with a clinically confirmed diagnosis of SMD using standardized measures. Most previous studies of health expenditure in people with mental health problems in LMICs have focused on depression/anxiety [7] or relied upon facility-based studies which are subject to selection bias [75]. The inclusion of matched comparisons without the condition in our study enabled us to estimate the net effect of SMD on out-of-pocket health expenditures. This approach is recommended by WHO in estimating economic consequences of a disease [76]. Furthermore, we estimated expenditures in a comprehensive and systematic way and used households as the preferred unit of analysis for assessing out-of-pocket payments because decisions about treatment and coping are based on negotiations within the household.

Limitations of the study are that self-reported household consumption expenditures may not be accurate due to recall bias. We did not include costs associated with treatment seeking, like transportation, which may lead to underestimation of the financial impact of out-of-pocket payments for health care on households. The study was based on a cross-sectional survey, so the temporal association between severe mental disorder and catastrophic expenditure was not established. Hence, future research could examine how OOP burdens vary over time with service utilization patterns.

## Conclusions

Catastrophic out-of-pocket health expenditures in households of a person with SMD at any threshold were high and associated with financial coping strategies which may lead to poorer health outcomes, entrenchment of poverty, and intergenerational disadvantage. Therefore, policy interventions aimed at addressing the high burden of OOP payments among vulnerable households should be in place. Such mechanisms can include: subsidies, cash transfers or exemption mechanisms for accessing health care and the scale up of integrated mental health care, as well as risk pooling mechanism such as community-based health insurance [77].

## Additional files

**Additional file 1: Figure S1.** Patient recruitment flow

**Additional file 2: Table S1.** Unadjusted odds of coping strategies for financial difficulties by mental health disorder and covariates.

## Data Availability

The data are being used for a Ph.D. student (YH) for his thesis and are not, therefore, available at the present time to the general public. The data may be requested from the corresponding author for verification of the analyses in this paper on a reasonable request.

## Abbreviations

Emerald: emerging mental health systems in low- and middle-income countries
GDP: gross domestic product
LMICs: low- and middle-income countries
OCRPIT: operational criteria checklist for psychotic illness and affective illness
OOP: out-of-pocket
PRIME: PRogramme for Improving Mental health carE
SAGE: Study on global Ageing and adult health
SMD: severe mental disorder
WHO: World Health Organization
WHODAS: WHO disability assessment schedule
