# Supplementary material for: Catastrophic out-of-pocket payments for households of people with severe mental disorder: a comparative study in rural Ethiopia
Source: Int J Ment Health Syst. 2019 Jun 1;13:39. doi: 10.1186/s13033-019-0294-7 (PMC6544918; doi:10.1186/s13033-019-0294-7)
Supplement: Supplementary file 1 — Additional file 1: Figure S1. Patient recruitment flow [file 13033_2019_294_MOESM1_ESM.docx]

Additional file 1: Figure S1: Patient recruitment flow
