# Supplementary material for: Catastrophic out-of-pocket payments for households of people with severe mental disorder: a comparative study in rural Ethiopia
Source: Int J Ment Health Syst. 2019 Jun 1;13:39. doi: 10.1186/s13033-019-0294-7 (PMC6544918; doi:10.1186/s13033-019-0294-7)
Supplement: Supplementary file 2 — Additional file 2: Table S1. Unadjusted odds of coping strategies for financial difficulties by mental health disorder and covariates. [file 13033_2019_294_MOESM2_ESM.docx]

Additional file 2: Table S1. Unadjusted odds of coping strategies for financial difficulties by mental health disorder and covariates.

| Characteristics | Total by subgroup | Coping strategies implemented for financial constraint | | | | | | | |
| --- | --- | --- | --- | --- | --- | --- | --- | --- | --- |
|  |  | Sold assets (n=238) | Drew up accounts at shops(n=97) | Cut down food consumption (n=125) | Withdrew children from school (n=65) | Relatives/family assistance (n=118) | Reduce medical visits (n=75) | Used savings (n=35) | Took on extra work (n=118) |
|  |  | COR(95%CI) | COR(95%CI) | COR(95%CI) | COR(95%CI) | COR(95%CI) | COR(95%CI) | COR(95%CI) | COR(95%CI) |
| HH of person with SMD | 241 | 0.4 (0.2,0.7)** | 1.0 (0.7,1.5) | 2.2 (1.5,3.1)*** | 4.1(2.4, 7.1)*** | 2.1(1.5,3.0)*** | 3.5 (2.2,5.5)*** | 0.9 (0.5,1.6) | 1.7(1.2,2.5)** |
| HH without person with SMD | 115 | 1.00^†^ | 1.00^†^ | 1.00^†^ | 1.00^†^ | 1.00^†^ | 1.00^†^ | 1.00^†^ | 1.00^†^ |
| Residence |  |  |  |  |  |  |  |  |  |
| Urban | 70 | 0.1(0.05,0.2)*** | 2.2(1.4,3.4)*** | 0.8 (0.5,1.3) | 0.8 (0.4,1.5) | 0.9(0.5,1.4) | 0.8 (0.4,1.4) | 0.8(0.3,1.6) | 1.4(0.9,2.1) |
| Rural | 286 | 1.00^†^ | 1.00^†^ | 1.00^†^ | 1.00^†^ | 1.00^†^ | 1.00^†^ | 1.00^†^ | 1.00^†^ |
| Gender |  |  |  |  |  |  |  |  |  |
| Male | 262 | 2.1(1.2,3.5)** | 0.5(0.3,0.9)* | 0.4(0.2,0.7)** | 0.9(0.5,1.8) | 1.0(0.6,1.8) | 0.4(0.2, 0.7)** | 1.4(0.6, 3.4) | 1.0(0.6, 1.7) |
| Female | 91 | 1.00^†^ | 1.00^†^ | 1.00^†^ | 1.00^†^ | 1.00^†^ | 1.00^†^ | 1.00^†^ | 1.00^†^ |
| Household consumption |  |  |  |  |  |  |  |  |  |
| Quintile 1 (lowest) | 58 | 1.1(0.5,2.2) | 1.3(0.7,2.4) | 3.1(1.7,5.6)*** | 1.7 (0.8,3.7) | 1.4 (0.8, 2.4) | 9.9 (3.7,26.1)*** | 0.9 (0.4,2.2) | 2.2(1.2,4.0)* |
| Quintile 2 | 63 | 0.5(0.3,1.1) | 1.2(0.6,2,2) | 2.4(1.3,4.4)** | 1.5 (0.7,3.2) | 1.2 (0.7,2.1) | 5.1(1.8,14.0)** | 0.8 (0.3,1.9) | 2.1(1.1,3.9)* |
| Quintile 3 | 75 | 0.8(0.4,1.6) | 1.1(0.5,2.0) | 2.2(1.6,5.1)*** | 1.5 (0.7,3.2) | 1.4 (0.8, 2.4) | 6.2 (2.3,16.8)*** | 0.7 (0.2,1.8) | 1.9(1.0,3.4)* |
| Quintile 4 | 74 | 0.7(0.6,2.5) | 1.0(0.8,1.8) | 1.6 (0.9,3.0) | 0.9 (0.4,2.0) | 1.1 (0.6,1.9) | 4.5 (1.6,12.5)** | 0.6 (0.1,1.9) | 1.6 (0.8,2.9) |
| Quintile 5(highest) | 86 | 1.00^†^ | 1.00^†^ | 1.00^†^ | 1.00^†^ | 1.00^†^ | 1.00^†^ | 1.00^†^ | 1.00^†^ |

* p<0.05, ** p<0.01, *** p<0.001; CI, 95% confidence interval; COR, crude odds ratio;*^†^ Reference group*; HH, household; The model was run separately for each coping strategy using the same set of independent variables
